# Engaging Isolated Hispanic and Latino Older Adult Caregivers in Remote Behavioral Interventions

**DOI:** 10.1093/geroni/igab046.494

**Published:** 2021-12-17

**Authors:** Caroline Silva

**Affiliations:** University of Rochester School of Medicine, Rochester, New York, United States

## Abstract

Recruiting Hispanic/Latino older adults for behavioral clinical trials is challenging, particularly those who are isolated. This presentation describes the recruitment process and feasibility/acceptability outcomes for the adaptation of two interventions (Connect for Caregivers and Engage Coaching) aimed at improving the social relationships and supports of older adult Hispanic/Latino caregivers of a family member with dementia. We compare online and community-based recruitment methods for English and Spanish-speaking Hispanic/Latino caregivers across the United States and present recruitment challenges during COVID-19. Of eligible participants, 82% were identified via online (e.g., research registry) and 18% via community-based (including snowball sampling) methods. Of participants recruited via online methods, 22% were Spanish-speaking, versus 100% of those recruited by community-based methods. Overall, 91% of all eligible/interested participants enrolled in at least one of the interventions. We discuss further the feasibility/acceptability of study procedures and the interventions, as the study was conducted remotely (via phone/Zoom) due to COVID-19.

